# Genome-Wide Sequence Analysis of Kaposi Sarcoma-Associated Herpesvirus Shows Diversification Driven by Recombination

**DOI:** 10.1093/infdis/jiy427

**Published:** 2018-07-14

**Authors:** Neneh Sallah, Anne L Palser, Simon J Watson, Nazzarena Labo, Gershim Asiki, Vickie Marshall, Robert Newton, Denise Whitby, Paul Kellam, Inês Barroso

**Affiliations:** 1 Wellcome Sanger Institute, Wellcome Genome Campus, Hinxton, Cambridge; 2 Kymab Ltd, Babraham Research Complex, Cambridge; 3 Medical Research Council/Uganda Virus Research Institute, Uganda Research Unit, London School of Hygiene and Tropical Medicine, United Kingdom; 4 Department of Medicine, Imperial College London, United Kingdom; 5 AIDS and Cancer Virus Program, Viral Oncology Section, Leidos Biomedical Research, Frederick National Laboratory for Cancer Research, Maryland

**Keywords:** virus, KSHV, comparative genomics, recombination, divergence

## Abstract

**Background:**

Kaposi sarcoma-associated herpesvirus (KSHV) establishes lifelong infection in the human host and has been associated with a variety of malignancies. KSHV displays striking geographic variation in prevalence, which is highest in sub-Saharan Africa. The current KSHV genome sequences available are all tumor cell line-derived or primary tumor-associated viruses, which have provided valuable insights into KSHV genetic diversity.

**Methods:**

Here, we sequenced 45 KSHV genomes from a Ugandan population cohort in which KSHV is endemic; these are the only genome sequences obtained from nondiseased individuals and of KSHV DNA isolated from saliva.

**Results:**

Population structure analysis, along with the 25 published genome sequences from other parts of the world, showed whole-genome variation, separating sequences and variation within the central genome contributing to clustering of genomes by geography. We reveal new evidence for the presence of intragenic recombination and multiple recombination events contributing to the divergence of genomes into at least 5 distinct types.

**Discussion:**

This study shows that large-scale genome-wide sequencing from clinical and epidemiological samples is necessary to capture the full extent of genetic diversity of KSHV, including recombination, and provides evidence to suggest a revision of KSHV genotype nomenclature.

## Teaser

This study presents 45 new KSHV genomes sequenced from Uganda, a KSHV endemic region. We present new evidence for the presence of inter- and intragenic recombination across genomes contributing to the diversification of KSHV strains into at least 5 types.

Kaposi sarcoma-associated herpesvirus (KSHV) also known as human herpesvirus-8 (HHV-8) was first discovered by Chang and colleagues in 1994 as the etiological agent of Kaposi sarcoma (KS) [1, 2]. KSHV-associated diseases predominantly occur in immunosuppressed individuals [3], with widespread human immunodeficiency virus (HIV) infection driving the KS epidemic, especially in sub-Saharan Africa. KSHV is also found to be associated with lymphoproliferative disorders, particularly primary effusion lymphoma (PEL) and multicentric Castleman disease [1, 4], and, more recently, KSHV inflammatory cytokine syndrome (KICS) in individuals with HIV coinfection [5]. Virus transmission is mainly via saliva [6, 7]; however, other modes of transmission have been reported [8].

Within 2 years of its discovery, the first KSHV genome sequence BC-1 was determined from a PEL cell line, revealing an approximately 165-kb dsDNA genome with an approximately 140-kb long unique coding region [9]. The KSHV genome map has changed little since its discovery, with the annotation of the GK18 sequence revealing 86 genes, of which 22 encode putative immunomodulatory proteins [9–11]. The KSHV genome shows high conservation with up to 99% sequence identity between viral strains; however, both 5′ and 3′ ends of the genome have higher sequence variability and as such have been used to characterize viral strains [12]. ORF K1 located at the 5′ termini of the genome encodes a highly glycosylated transmembrane protein, with hypervariable regions (V1 and V2) with up to 30% amino acid variability, resulting in 7 major K1 subtypes, A–E and more recently F [12–15]. The P (predominant), M (minor), and N genotypes of KSHV arise from the K15 gene at the 3′ termini of the genome, a gene that encodes an integral membrane protein with up to 30% sequence identity at the amino acid level [16–18]. While the central region of the KSHV genome is highly conserved, 9 discrete loci with lower levels of variation compared to K1 and K15 have also been used in a number of phylogenetic studies for subtype characterization [18].

Prior to recent developments in next-generation sequencing technologies, large scale whole-genome comparisons were not feasible. A recent study, conducted by Olp and colleagues sequenced 16 whole genomes directly from skin lesions of Zambian KS patients [19]. They showed that low-level genetic variation in the central conserved genome region contributes to a unique phylogenetic structure, with distinct genomic variants from Zambian isolates compared to Western (United States and Greece) isolates [19]. These types of genome-wide sequencing studies allow us to account for the remaining >90% of the genome to further understand the diversity of KSHV at the whole-genome level.

With the current genome sequences from only 4 countries (United States [9, 20–22], Greece [10], Zambia [19] and most recently Japan [23]), and all from individuals with KSHV-associated diseases, we are now beginning to understand the extent of KSHV genomic diversity. While very important ground work has been laid by these previous studies, no genomes have been obtained from asymptomatic persistently infected individuals and thus nontumor-associated KSHV genomes have never been characterized. Here, we performed whole-genome sequencing of KSHV from saliva of Ugandan individuals free of KSHV-associated disease, and assessed the variability between KSHV sequences isolated from different sources, including diseased individuals and patients from diverse geographic origins. In addition, we explored the presence of inter- and intragenotype recombination within the Uganda General Population Cohort (GPC) [24] and in a wider context.

Uganda is a good country to study molecular epidemiology and phylogeography of KSHV, as it is inhabited by different ethnolinguistic groups with divergent historic origins as a result of migration over several hundred years from surrounding regions [25]. In addition, the population has the highest reported seroprevalence of KSHV in the world [26–28]. In the GPC, the seroprevalence of KSHV is >90%. Several studies conducted in Uganda have provided valuable insights into KSHV seroepidemiology and transmission [6, 7, 29–32], therefore characterizing genetic diversity on a genome-wide level will provide invaluable insights to further our understanding of KSHV diversity and its evolution.

## METHODS

### Sample Collection and Ethics

The GPC is a population-based cohort in rural south-west Uganda consisting of 25 neighboring villages mainly inhabited by peasant farmers [24]. Households are scattered with some concentrated in the trading centers. Saliva samples (N = 2036) were collected from asymptomatic individuals during medical survey round 24 between January and July 2015. Saliva (2 mL) was collected with the Oragene DNA self-collection kit, OMNIgene ORAL OM-505 (DNA Genotek, ON, Canada) following manufacturer’s instructions and stored at −80°C prior to shipment on dry ice to the Sanger Institute. Informed consent was obtained for genetic testing from participants either with signature or a thumb print if the individual was unable to write. The study was approved by the Uganda Virus Research Institutes, Research Ethics Committee (Ref. GC/127/10/10/25), the Uganda National Council for Science and Technology, and the UK National Research Ethics Service, Research Ethics Committee (Ref.11/H0305/5).

### DNA Extraction and Purification

All sample preparation was performed in class II biosafety cabinets using aseptic techniques. Saliva samples were lysed and RNA removed with proteinase K (600 mAU/mL) Buffer VXL solution and RNase A (100 mg/mL) treatment (Qiagen, UK). Aliquots of lysates (200 μL) were then extracted using the QIAamp 96 DNA QIAcube HT robot following the manufacturer’s protocol, and the remainder stored at −80°C.

### Viral DNA Quantification

Quantitative polymerase chain reaction (qPCR) targeting the KSHV ORF73 gene was used for viral genome detection and determination of viral genome load. This was measured by determining the viral copy number relative to a 10-fold dilution of control BCBL-1 DNA against a standard curve with a detection range of 3 × 10^6^–10 copies/mL (cycle threshold [Ct] of 15 to 43). Out of the 2036 samples, 746 were processed in duplicates using the QuantiTect Muliplex PCR kit (Qiagen, UK) on a Stratagene Mx3005P (Agilent Technologies, UK). Primers and probes targeting ORF73 (Metabion international AG, Germany) were designed for viral detection using sequences from Lallemand et al [33]. Glyceraldehyde-3-phosphate dehydrogenase was used to assess DNA quality with sequences from Pardieu et al [34]. Primer-probe mixes were diluted to a 20× solution and following the qPCR conditions from Lallemand et al [33]. Data analysis was performed using MxPro v4.10 qPCR software (Agilent Technologies).

### KSHV Whole-Genome Sequencing

The low abundance of viral DNA compared to the host DNA, along with the large KSHV genome, makes sequencing of KSHV quite challenging, therefore, in this study we sequenced whole genomes from 244 samples with detectable viral DNA (Ct values < 36) using the SureSelect method (version 1.1; Agilent Technologies) [35]. Excluding repeat regions, baits were designed to include all published KSHV genomes sequenced from PELs (BCBL-1, BC-1, JSC-1, VG-1). Samples were multiplexed on an 8-lane flow cell with 24 samples per lane; cluster generation and sequencing was performed on an Illumina HiSeq 2500 sequencer. Sequencing reads were 250-bp paired ends in FASTQ format with per base Phred quality scores.

### Guided Assembly of KSHV Whole Genomes

The QUASR QC pipeline (http://sourceforge.net/projects/quasr) [36] was used to retain high-quality full-length reads. Duplicate reads and paired reads with a raw median Phred quality score Q < 32 were either filtered out or trimmed from the 3′ end until Q > 32, reads less than 100 bp post trimming were also excluded. High-quality paired-end reads post-QC were then mapped back to GK18 and BC1 reference sequences using Burrows-Wheeler Aligner (BWA) [37] and the depth and coverage calculated using SAMTools [38]. Pairwise-correlation was calculated using Pearson correlation in R for qPCR viral load, KSHV mapped reads (%), and sequencing depth.

### Comparative Sequence and Population Structure Analysis

For comparative sequence analysis we selected sequences from 45 Ugandan individuals with an average sequencing depth of >20×, and aligned them with 25 publicly available KSHV genomes using MAFFT [39] (v7.0) and viewed them using AliView software. Repeat regions across the alignment were masked with coordinates retrieved from the GK18 reference sequence annotation in Genbank (NC_009333). Genome-wide mutations relative to the GK18 reference sequence were visualized in a 1000-nucleotide (nt) scanning window. Principal components analysis (PCA) was performed on all single-nucleotide polymorphisms (SNPs) in the genomes using the scikit-learn package implemented in ScientificPython. Phylogenetic analysis was performed following alignment of the coding sequences of the K15 gene and K1 gene along with representative sequences (Supplementary Table 1). The alignments were used to infer trees using maximum-likelihood methods implemented in RAxML (v8) with 1000 bootstrap replicates under a general time reversible model of nucleotide substitution and including a Gamma distribution for among site rate variation [40].

### Recombination Analysis

To identify conflicting phylogenetic signals, Neighbor-net split networks were constructed with SplitsTree 4.14 [41], using the Uncorrected_P characters’ transformation and excluding gap sites. To calculate a measure of statistical significance for recombination we used the phi test, which has been proven to give reliable results for conserved DNA sequences [42]. To identify recombination and potential breakpoints we used Kishino Hasegawa test (*P* < .05) and the Akaike information criterion for goodness of fit implemented by the Genetic Algorithm Recombination Detection program (GARD) [43]. Further statistical support were provided by using the algorithms implemented in RDP4 suite [44] with default settings. Bootscan analysis was also performed across the genomes using the SimPlot program [45] with a window size of 4000 nt and a 1000 nt step size with a bootstrap cutoff of 70%.

### Data Deposition

The sequencing data from this study has been submitted to the European Nucleotide Archive (http://www.ebi.ac.uk/ena) under the accession numbers listed in Table 1.

**Table 1.** Characteristics of Samples Used in This Study

Name	Origin	K15 Type	K1 Type	New Type^a^	Sample Source	HIV Status^b^	Clinical Presentation	Accession Number	Ref.
UG110	Uganda	P	A5	P1	Saliva	0	Asymptomatic	ERS1615765	…
UG114	Uganda	P	A5	P2	Saliva	0	Asymptomatic	ERS1615766	…
UG117	Uganda	P	C1	P2	Saliva	0	Asymptomatic	ERS1615774	…
UG118	Uganda	M	B1	M1	Saliva	0	Asymptomatic	ERS1615777	…
UG119	Uganda	M	A5	M1	Saliva	0	Asymptomatic	ERS1615780	…
UG12	Uganda	P	B1	P1	Saliva	1	HIV + asymptomatic	ERS1615738	…
UG120	Uganda	P	B1	P2	Saliva	0	Asymptomatic	ERS1615783	…
UG125	Uganda	P	B1	P1	Saliva	0	Asymptomatic	ERS1615800	…
UG126	Uganda	P	B1	P1	Saliva	0	Asymptomatic	ERS1615707	…
UG128	Uganda	P	A5	P1	Saliva	0	Asymptomatic	ERS1615712	…
UG129^c^	Uganda	P	B1	P1	Saliva	0	Asymptomatic	ERS1615715	…
UG13	Uganda	P	B1	P1	Saliva	0	Asymptomatic	ERS1615741	…
UG131^c^	Uganda	M	B1	M2	Saliva	0	Asymptomatic	ERS1615723	…
UG132	Uganda	P	A5	P2	Saliva	0	Asymptomatic	ERS1615725	…
UG133	Uganda	P	B1	P1	Saliva	0	Asymptomatic	ERS1615727	…
UG134	Uganda	P	A5	P1	Saliva	0	Asymptomatic	ERS1615730	…
UG136	Uganda	P	B3	P2	Saliva	0	Asymptomatic	ERS1615737	…
UG137	Uganda	P	A5	P1	Saliva	0	Asymptomatic	ERS1615744	…
UG141	Uganda	P	B1	P1	Saliva	0	Asymptomatic	ERS1615761	…
UG145	Uganda	M	A5	M1	Saliva	0	Asymptomatic	ERS1615775	…
UG146	Uganda	P	A5	P1	Saliva	0	Asymptomatic	ERS1615778	…
UG148	Uganda	P	A5	P1	Saliva	0	Asymptomatic	ERS1615784	…
UG149	Uganda	P	A5	P2	Saliva	0	Asymptomatic	ERS1615786	…
UG15	Uganda	M	A5	M1	Saliva	0	Asymptomatic	ERS1615748	…
UG151	Uganda	P	B1	P1	Saliva	0	Asymptomatic	ERS1615793	…
UG152	Uganda	P	A5	P1	Saliva	0	Asymptomatic	ERS1615795	…
UG155	Uganda	P	B1	P2	Saliva	0	Asymptomatic	ERS1615706	…
UG156	Uganda	P	C1	P1	Saliva	1	HIV + asymptomatic	ERS1615711	…
UG157	Uganda	M	C1	M1	Saliva	1	HIV + asymptomatic	ERS1615714	…
UG158	Uganda	P	A5	P1	Saliva	0	Asymptomatic	ERS1615719	…
UG159	Uganda	P	A5	P1	Saliva	0	Asymptomatic	ERS1615721	…
UG16	Uganda	M	A5	M1	Saliva	1	HIV + asymptomatic	ERS1615752	…
UG160	Uganda	M	B1	M2	Saliva	0	Asymptomatic	ERS1615722	…
UG162	Uganda	P	B1	P1	Saliva	0	Asymptomatic	ERS1615729	…
UG163	Uganda	P	B1	P1	Saliva	0	Asymptomatic	ERS1615732	…
UG164	Uganda	P	A5	P2	Saliva	0	Asymptomatic	ERS1615736	…
UG165	Uganda	P	A5	P1	Saliva	0	Asymptomatic	ERS1615740	…
UG166	Uganda	P	A5	P1	Saliva	0	Asymptomatic	ERS1615743	…
UG168	Uganda	P	B1	P1	Saliva	1	HIV + asymptomatic	ERS1615750	…
UG212	Uganda	P	A5	P1	Saliva	0	Asymptomatic	ERS1615837	…
UG219	Uganda	M	B4	M1	Saliva	0	Asymptomatic	ERS1615860	…
UG222	Uganda	P	A5	P1	Saliva	0	Asymptomatic	ERS1615807	…
UG226	Uganda	P	A5	P1	Saliva	1	HIV + asymptomatic	ERS1615813	…
UG237	Uganda	M	B4	M2	Saliva	0	Asymptomatic	ERS1615851	…
UG244	Uganda	P	A5	P1	Saliva	0	Asymptomatic	ERS1615861	…
BC1	United States	M	A2	M1	B-cell line	0	PEL	U75698.1	[9]
BCBL1	United States	P	A3	P1	B-cell line	0	PEL	HQ404500.1	[20]
DG1	United States	P	A5	P1	Blood	0	KICS	JQ619843.1	[22]
GK18	Greece	P	C3	P1	KS biopsy	1	Classic KS	AF148805.2	[10]
JSC1	United States	P	C3	P1	B-cell line	0	EBV + PEL	GQ994935.1	[21]
ZM004	Zambia	P	B1	?	KS biopsy	1	Classic KS	KT271453	[19]
ZM027	Zambia	P	B1	P2	KS biopsy	1	Classic KS	KT271454	[19]
ZM091	Zambia	P	A5	P2	KS biopsy	1	Classic KS	KT271455	[19]
ZM095	Zambia	N	B4	N	KS biopsy	1	Classic KS	KT271456	[19]
ZM102	Zambia	P	B4	P2	KS biopsy	1	Classic KS	KT271457	[19]
ZM106	Zambia	P	B1	P1	KS biopsy	1	Classic KS	KT271458	[19]
ZM108	Zambia	P	B4	P2	KS biopsy	1	Classic KS	KT271459	[19]
ZM114	Zambia	P	B3	P2	KS biopsy	1	Classic KS	KT271460	[19]
ZM116	Zambia	P	B4	P1	KS biopsy	1	Classic KS	KT271461	[19]
ZM117	Zambia	P	B4	P2	KS biopsy	1	Classic KS	KT271462	[19]
ZM118	Zambia	P	B1	P1	KS biopsy	1	Classic KS	KT271463	[19]
ZM121	Zambia	P	B1	P2	KS biopsy	1	Classic KS	KT271464	[19]
ZM123	Zambia	P	B1	P1	KS biopsy	1	Classic KS	KT271465	[19]
ZM124	Zambia	P	B1	P1	KS biopsy	1	Classic KS	KT271466	[19]
ZM128	Zambia	N	B1	N	KS biopsy	1	Classic KS	KT271467	[19]
ZM130	Zambia	P	B3	P2	KS biopsy	1	Classic KS	KT271468	[19]
Japan1	Japan	M	C3	M1	Cell line	0	Non-AIDS KS	LC200589	[23]
Miyako1	Japan	M	C3	M1	KS biopsy	0	Non-AIDS KS	LC200586	[23]
Miyako2	Japan	M	C3	M1	KS biopsy	0	Non-AIDS KS	LC200587	[23]
Miyako3	Japan	M	C3	M1	KS biopsy	0	Non-AIDS KS	LC200588	[23]

Abbreviations: EBV, Epstein-Barr virus; KICS, Kaposi sarcoma-associated herpesvirus inflammatory cytokine syndrome; KS, Kaposi sarcoma; PEL, primary effusion lymphoma.

^a^Based on whole-genome data.

^b^HIV status: 0, negative; 1, positive.

^c^Belong to the same household.

## RESULTS

### Characteristics of Samples Used in This Study

We sequenced 244 out of 746 (32.7%) Ugandan samples with detectable KSHV viral load (range: 5.35 × 10^5^ to 1.5 copies/mL). Viral load was strongly positively correlated with the percentage of mapped reads (*r*^2^ = 0.84) and a high viral load was positively correlated with achieving good (ie, >20-fold) mean sequencing depth with >90% coverage across the genome. Out of the 244 samples, 45 (18.4%) had > 20-fold coverage per genome nucleotide and these corresponded to the samples with the highest viral loads (10^4^–10^5^ copies/mL). They were collected from 8 neighboring villages (12–19) in the GPC study area [24], consisting of 21 men and 24 women between the ages of 16 and 86 years (mean ± S.D, 41.65 ± 20.69). Five individuals were also HIV positive. Sample details for all 45 new GPC genome sequences are presented in Supplementary Table 2.

### KSHV Genome Variability Analysis

**Figure 1.**
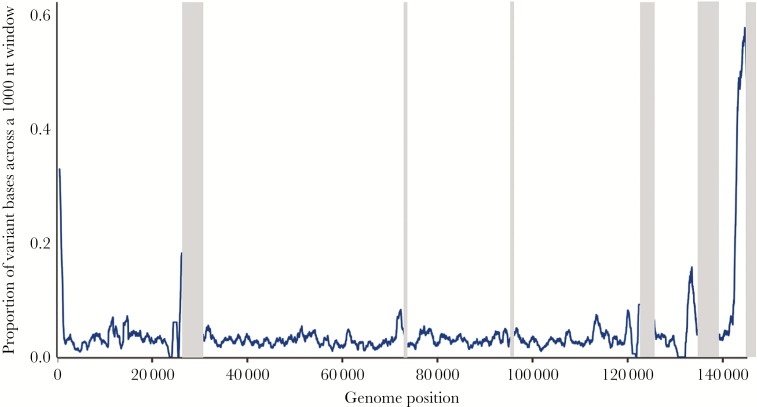
Whole-genome variation across 70 genomes. Line graph plotted across the genome showing the proportion of variant bases in a 1000-nucleotide (nt) sliding window where at least 1 Kaposi sarcoma-associated herpesvirus genome sequence has a single nucleotide polymorphism relative to the GK18 reference sequence. Grey bars indicate masked-out repeat regions.

To determine how variable the 45 GPC KSHV genomes were and explore which parts of the genome were contributing to the most variation, we performed a multiple sequence alignment including the 25 previously published KSHV genome sequences from (Table 1). The proportion of variants to the GK18 reference sequence were determined within a 1000-nucleotide sliding window. Consistent with previous findings, this showed approximately 35% of all nucleotide substitutions were at the 5′ end of the genome, which corresponds to the K1 gene, and approximately 60% were at the 3′ end of the genome, corresponding to the K15 gene, with modest variation observed across the central regions of the genome (Figure 1). The total number of SNPs between each of the KSHV genomes can be seen in detail in Supplementary Figure 1 ordered by sequence identity to GK18.

### Virus Genome-Wide Population Structure Analysis

**Figure 2.**
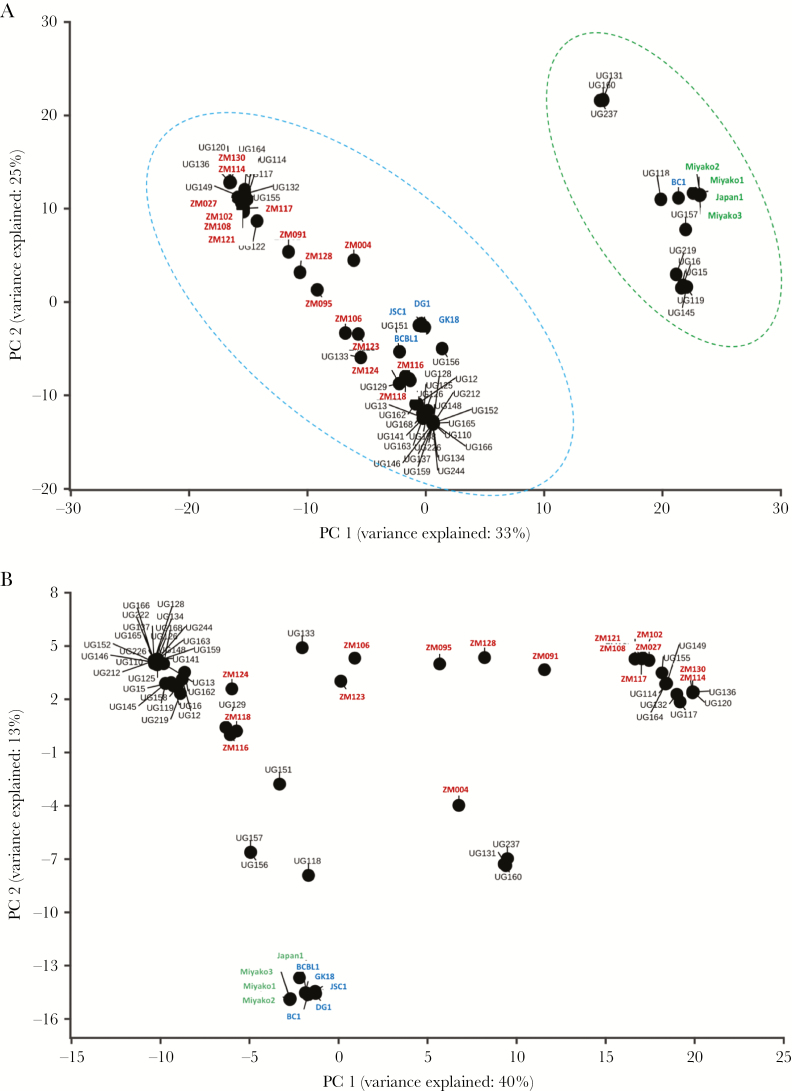
Principal component analysis (PCA) separates strains by type and geographic origin. PCA of all Kaposi sarcoma-associated herpesvirus (KSHV) strains (45 new strains and 25 published strains) based on single-nucleotide polymorphisms relative to the GK18 reference sequence in a full-genome multiple-sequence alignment. *A*, Principal component 1 (PC 1) separates all strains based on type P (blue dotted circle) and type M (green dotted circle). *B*, PCA of central genome minus variable K1 and K15 genes shows some geographic clustering of KSHV strains, with separation of the African strains (black and red) compared to Western (blue) and Japanese (green).

To investigate the population structure of the 45 new genomes from Uganda in a wider context, we performed a PCA along with the 25 previously published strains. PC1 separated strains clearly into the 2 distinct types (Figure 2A), which have been previously classified as the type P and type M strains based on variation in the K15 gene and was the greatest contributor to the variance observed (32%). In addition, within each type, the Western samples (ie, GK18, BCBL-1, JSC-1, and DG-1) and Japanese samples cluster separately from the African samples (ie, Zambia and Uganda) and neither showed separation by country on PC2. We observed no distinct clustering of samples by strain in the respective villages. In addition, the PCA showed no distinct clustering patterns between genomes isolated from saliva compared to other sources, or between samples from asymptomatic versus diseased.

To assess the contribution of K1 and K15 genes to population structure, we realigned the genomes of all the samples excluding the K1 and K15 genes and re-examined the PCA. While the clustering by type (P vs M) was lost, geographical clustering was observed with the Western and Japanese genomes clustered together and away from the African genomes (Figure 2B). This showed that genes in the central region were the major contributors to the geographical clustering observed.

### Genotypic Diversity of Strains in the GPC

Because the K1 and K15 genes have been used for virus type classification we generated trees for each gene to determine the genotypes circulating in the 45 Ugandan samples. For the K15 phylogenetic analysis, clear separation was observed between the strains types P and M; most Ugandan samples (78%) grouped with the type P strain, while 22% of samples grouped with the type M, and none of the GPC samples belonged to the type N strain (Supplemental Figure 2 and Table 1). The major types observed in the K15 phylogenetic tree are also consistent with the clustering identified in the PCA (Figure 2A)

For the K1 phylogenetic analysis, we aligned the 70 genomes with K1 genes for the previously described genotypes (Supplementary Table 1). Of the 45 Ugandan GPC samples, 40% grouped with B genotypes, 53% grouped with the A genotype, and 7% grouped with the C genotype (Supplemental Figure 3 and Table 1). While the B genotypes displayed heterogeneity in subtypes, clustering mainly with B1 and B3, all the A genotypes grouped with the A5 subtype.

### Recombination Analysis

**Figure 3.**
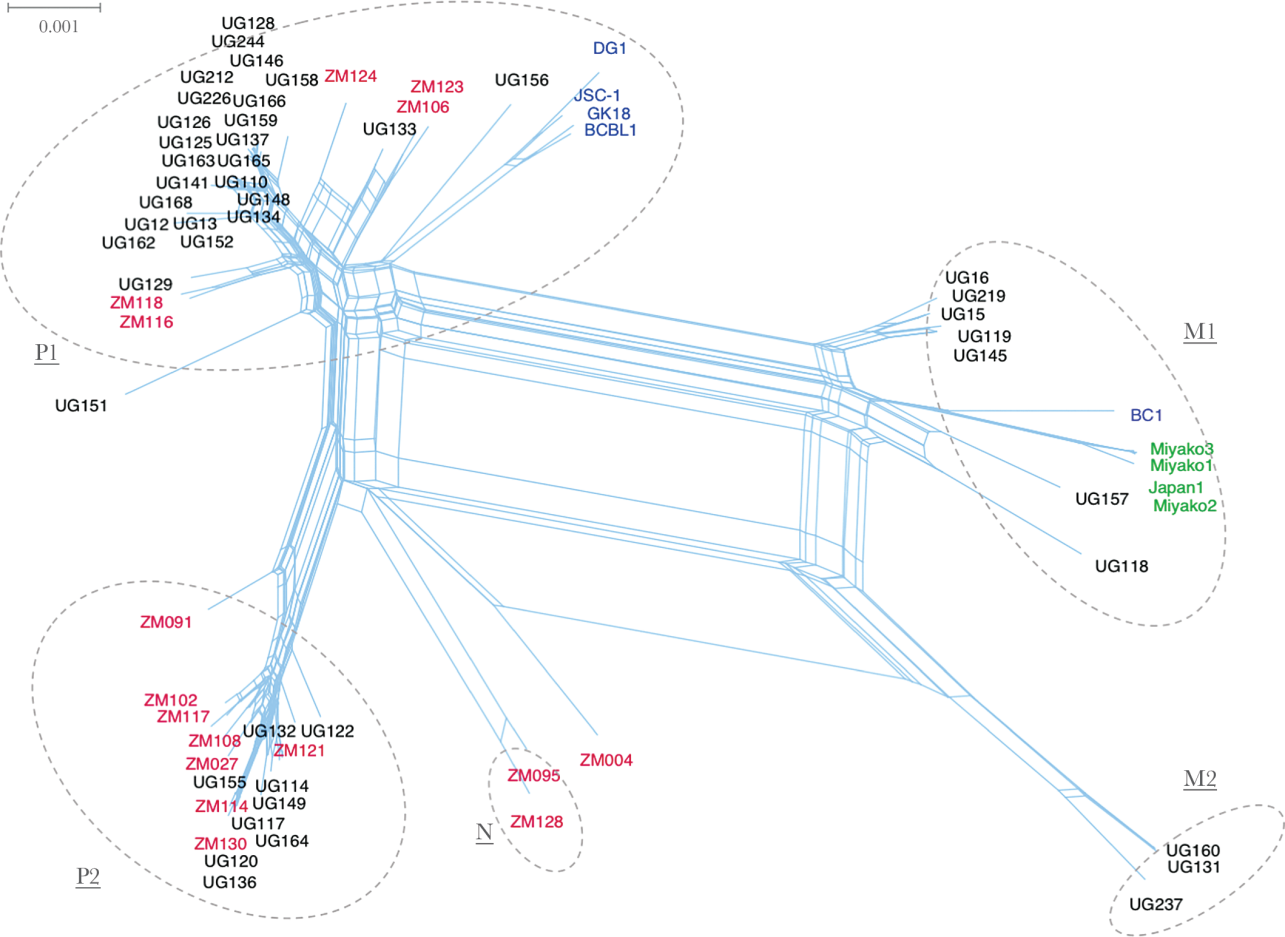
Neighbor-net phylogenetic network based on complete genomes of all 70 Kaposi sarcoma-associated herpesvirus samples. Parallel edges depict conflicting phylogenetic signals. The samples clustered into 5 distinct types (dotted circles) designated types P1, P2, M1, M2, and N, and 1 outlier ZM004, which did not cluster well with any type and thus may be a putative intertype recombinant or a novel type. The tips are labeled to represent sample geographic origin: black (Uganda), red, (Zambia), blue (Western), and green (Japan).

To identify conflicting phylogenetic signals representative of evolutionary splits potentially due to recombination, we generated a split network based on all 70 genomes. Phylogenetic incongruences were illustrated by parallel internal branches, which is typical when there are several recombination events and/or convergent evolution. This analysis revealed the subgrouping of samples into 5 potentially distinct types labeled P1, P2, M1, M2, and N (Figure 3), as opposed to the original K15 classification into only 3 types: P, M, and N (Supplementary Figure 2), and 1 outlier, ZM004, which could be either a potential recombinant or novel type that has not been well sampled. A phi test provided strong statistical evidence of recombination (*P* < .00001) over convergent evolution. Furthermore, a neighbor-net split network of the genomes minus the K1 and K15 genes also show strong evidence of recombination (Supplementary Figure 4) and highlight the contribution of central parts of the genome to diversity and population structure (similarly to the PCA in Figure 2B).

Further resolving the extent of recombination events of KSHV is nontrivial owing to high conservation of KSHV genomes. Topological incongruences were identified by examining the trees generated by GARD either side of the confirmed breakpoint and using the phi test (*P* < .05) and RDP4 (>2 methods) we confirmed significant evidence of intragenic recombination within 8 (K1, ORF4, ORF6, ORF9, ORF11, ORF21, ORF48, and ORF64) (Table 2) out of 86 genes that potentially contribute to inter- and intratype recombination that are present across genomes.

**Table 2.** Kaposi Sarcoma-Associated Herpesvirus Genes With Evidence of Recombination

Gene	GARDa	Phi test	RDP4	Breakpoint (Bootstrap Support)	Alignment length, Codons
K1	Yes	Yes	Yes	362(0.71)	867
ORF4	Yes	Yes	Yes	475 (0.86)	1650
ORF6	Yes	Yes	No	1622 (0.18)	3399
ORF9	Yes	Yes	Yes	408 (0.99)	3036
ORF11	Yes	Yes	Yes	462 (0.51)	1221
ORF21	Yes	Yes	Yes	931 (0.68)	1740
ORF48	Yes	Yes	No	909 (0.95)	1206
ORF64	Yes	Yes	Yes	6561 (0.35)	7905

Abbreviations: AICc, Akaike information criterion; GARD, genetic algorithm recombination detection; KH, Kishino Hasegawa; ORF, open reading frame.

^a^GARD: both AICc and KH significant, Phi-test: *P* < .05, RDP4: breakpoints with *P* < .05 and supported by at least 2 methods (RDP, GENECONV, MAXCHI, CHIMAERA, BOOTSCAN, SISCAN, 3SEQ).

**Figure 4.**
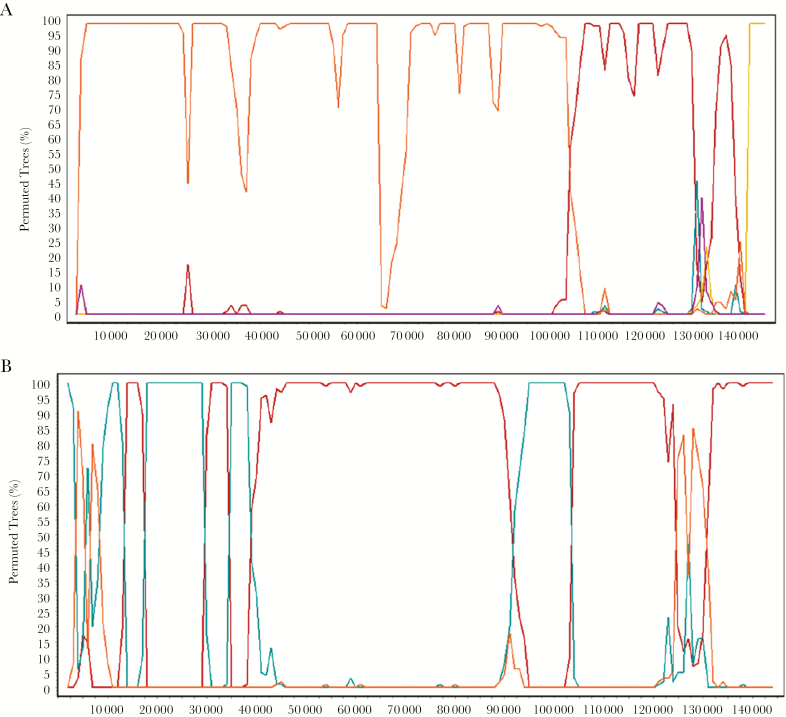
Evidence for recombination between different Kaposi sarcoma-associated herpesvirus types. *A*, Intertypic recombinant type M2. Bootscan plot comparing type M2 query with consensus sequence of types P2 (red), P1(green), M1(yellow), N (purple), and ZM004 (orange). *B*, Intratypic recombinant ZM091. Bootscan plot comparing ZM091 with consensus sequences of types P2 (red), P1(green), and ZM004 (orange). Shifting phylogenetic signals and fragmented genomes are indicative of the presence of recombination.

To analyze the degree of genome fragmentation and visualize potentially shifting phylogenetic relationships, we generated a consensus sequence for each of the 5 types (P1, P2, M1, M2, and N) and, along with the ZM004 outlier genome, performed bootscan analyses using Simplot and statistical support provided by the RDP4 suite. These analyses showed strong support for intertypic recombination as displayed by the fragmentation across the genomes, suggestive of multiple recombination events over time (Figure 4A and Supplementary Figure 5). Particularly, the type M2 genomes, which show high identity (few SNPs) with ZM004, displayed conflicting phylogenetic signals with 2 recombination breakpoints (Figure 4A). As shown in the bootscan plot, clustering of the type M2 with ZM004 changes at approximate positions 105000 and 140000, substantiating the presence of recombination with a type P2 genome and type M1 genome, respectively (Figure 4A). Split networks showed conflicting phylogenetic signals within each type, with the phi test showing statistically significant evidence of recombination (*P* < .05), which was confirmed by bootscan analysis in Simplot. For example, ZM091 a strain and outlier in the Neighbor-net tree (Figure 3) and potentially belonging to the P2 type, showed multiple recombination crossovers (Figure 4B).

## DISCUSSION

Genome-wide sequence analyses of viruses have become central to enhancing our understanding of the biology and epidemiology of viruses. Previous genetic analyses of KSHV genomes generated from KS, PEL, and KICS samples have provided invaluable insights into KSHV genomic architecture [9, 10, 19, 22], and laid the groundwork for comparative genomics; however, they may not be representative of those found in the general disease-free population. Here, we performed a comparative genomic variation and recombination analyses of the 45 new KSHV genomes, together with 25 previously published genomes from Greece, United States, Zambia, and Japan (Table 1) and present novel evidence of pervasive recombination throughout the genome.

Studies using saliva pose a significant challenge for virus genome sequencing given the virus is difficult to detect, particularly in asymptomatic individuals unless they are actively shedding virus. For oncogenic herpesvirus, viral levels are much lower in saliva and blood compared to tumor biopsies or tumor-derived cell lines, which may explain the paucity of virus genomes from healthy individuals. We and others have used virus genome capture and sequencing to sequence varicella zoster virus (VZV) [46], Epstein-Barr virus (EBV) [35, 47], HCMV [48], and now KSHV [19]. Our threshold for high-quality KSHV sequencing by target capture of >10^4^ genome copies/mL allowed us to derive hitherto unobtainable genome-wide sequences.

While the type P/type M classification, based on variation in the K15 gene, remained the major form of variation correlating with whole-genome clustering, the central genome region contributed to geographic clustering of samples in this study, consistent with the Zambian KS study [19]. Geographic association of K1 genotypes has been reported by several studies globally and our findings are consistent with previous studies of African strains; however, with intragenic recombination occurring in K1, phylogenetic relationships previously described may not be accurate. The heterogeneous distribution of KSHV genotypes throughout all villages, suggesting cross-village transmission, is not surprising given how well connected the villages are, with relaxed administrative boundaries enabling ease of access and movement between villages [24]. However, to reliably identify transmission patterns in this study, more household samples across age groups would be required.

Most strikingly, we observe strong statistical evidence of multiple recombination events across the KSHV genome leading to the grouping of samples into at least 5 distinct types. While these types broadly reflect the population structure associated with the K15 gene, it is evident that the genotyping based on the K15 gene along with the K1 gene does not robustly capture genetic diversity. While previous KSHV studies have reported the presence of genetic recombinants driven by multiple recombination events [16, 28], breakpoints could not be accurately assessed because the distances between the genes sequenced were too great. Here, we identify 8 genes with recombination and show evidence of shifting phylogenetic signals with recombination crossovers present throughout the KSHV genomes suggestive of pervasive recombination and potentially novel types, consistent with other examples occurring in EBV, herpes simplex virus (HSV), and VZV genomes [46, 47]. The KSHV genome is more conserved than those of EBV, VZV, HSV-1, and HSV-2, therefore it is challenging to accurately infer recombination between similar parental strains or classify them with statistical confidence. The extent of recombination and the extreme differences in SNP density between the K1 and K15 genes compared to the central genes make phylogenetic relationships of large regions of the genome difficult to interpret and prevent the accurate identification of the ancestry of strains at this stage. Recombination suggests that at a particular time reinfection or simultaneous coinfection by multiple strains occurred in single cells of an individual; however, disentangling intrahost genomic diversity as a result of mixed infections or reinfections from on-going evolution is nontrivial, particularly for a virus with such high identity across strains. It is unclear when, where, and how this process occurs as this requires prior knowledge about the parental strains involved in the process, which we cannot ascertain in this study. While we cannot exclude the possibility of mixed infections, to robustly confirm the distribution of genomic mixtures, multiple samples for the same individuals across time and/or compartments [49] would be advantageous.

In conclusion, recombination across the KSHV genome contributes to the divergence of 5 proposed distinct types, designated here P1, P2, M1, M2, and N, and 1 potentially novel type that has not been well sampled, including the sole genome ZM004. Given that these data are based on the analyses of 70 genomes, it is likely that the recombination events reported here are still an underestimation. Therefore, given our limitations, greater sampling depth from other parts of the world providing a more comprehensive global dataset, would be essential to examine the full extent of recombination in KSHV genomes. We speculate that certain types may contribute to virus transmissibility, which could be a very important contributing factor for why Uganda sustains such a high KSHV prevalence compared to other parts of the world. It would be essential to investigate the functional consequences of these new types on viral pathogenesis. Furthermore, the existence of such recombination should be considered in any phylogenetic analysis of KSHV sequence data, and viral characterization based on whole-genome diversity needs to be considered coupled with a reassessment of the nomenclature to accurately classify genotypes.

## Supplementary Data

Supplementary materials are available at *The Journal of Infectious Diseases* online. Consisting of data provided by the authors to benefit the reader, the posted materials are not copyedited and are the sole responsibility of the authors, so questions or comments should be addressed to the corresponding author.

Supplementary Figure 1

Supplementary Figure 2

Supplementary Figure 3

Supplementary Figure 4

Supplementary Figure5

Supplementary Table 1

Supplementary Table 2

## Notes

***Acknowledgments.*** We thank all participants who contributed to this study, Matt Cotten for designing the RNA baits, and the sequencing teams at the Wellcome Sanger Institute for sequencing the samples.

***Financial support.*** This work was supported by the UK Medical Research Council (MRC) and the UK Department for International Development (DFID) under the MRC/DFID Concordat Agreement; with further support from the Wellcome Trust (grant numbers WT098051 and WT090132), and the National Cancer Institute, National Institutes of Health (contract number HHSN261200800001E).

***Potential conflicts of interest.*** All authors: No reported conflicts of interest. All authors have submitted the ICMJE Form for Disclosure of Potential Conflicts of Interest. Conflicts that the editors consider relevant to the content of the manuscript have been disclosed.
