# Supplementary figures and images for: Genome-Wide Sequence Analysis of Kaposi Sarcoma-Associated Herpesvirus Shows Diversification Driven by Recombination
Source: J Infect Dis. 2018 Jul 14;218(11):1700–10. doi: 10.1093/infdis/jiy427 (PMC6195662; doi:10.1093/infdis/jiy427)

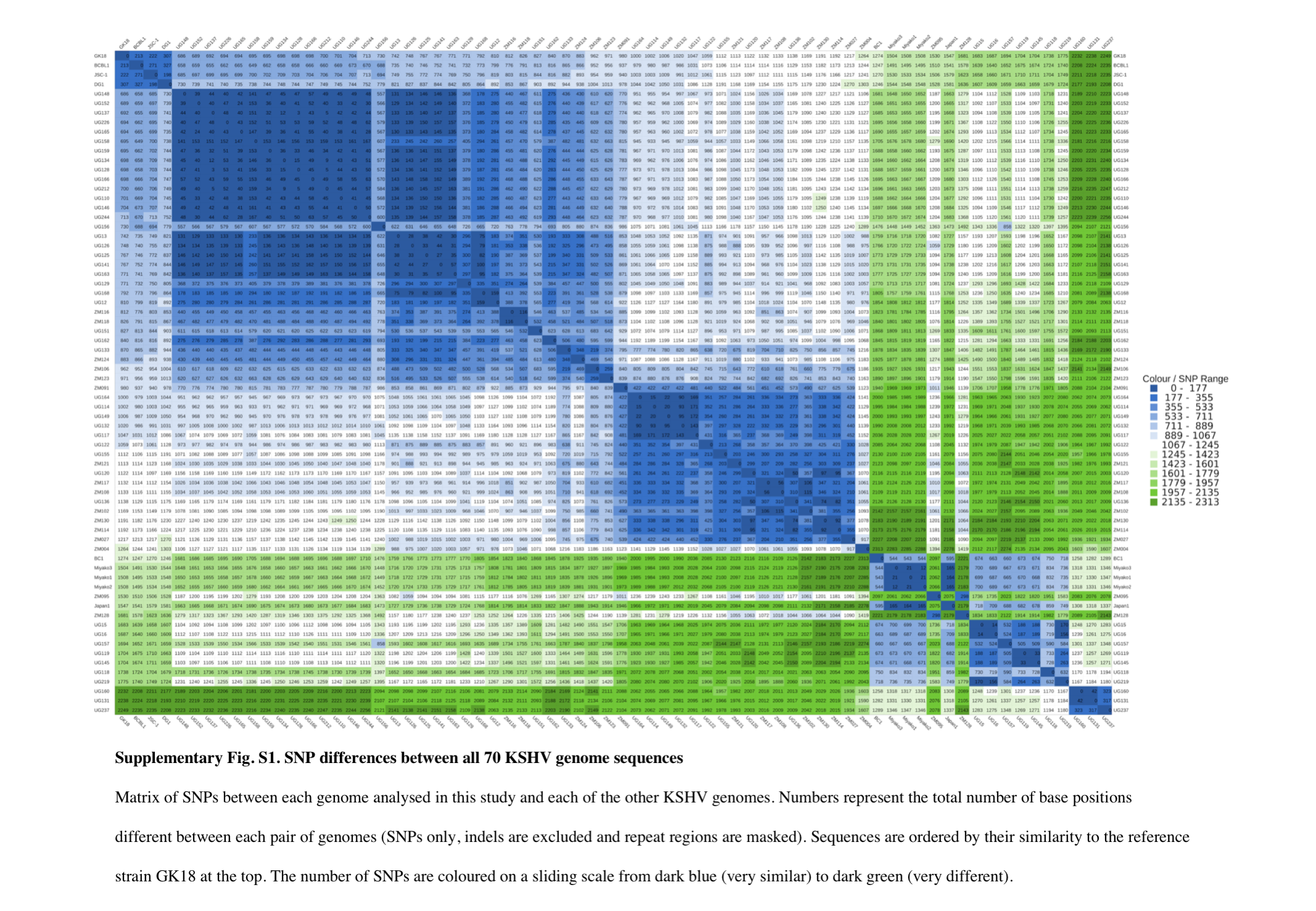

Supplement: Supplementary Figure 1 [file jiy427_suppl_supplementary_figure1.png]

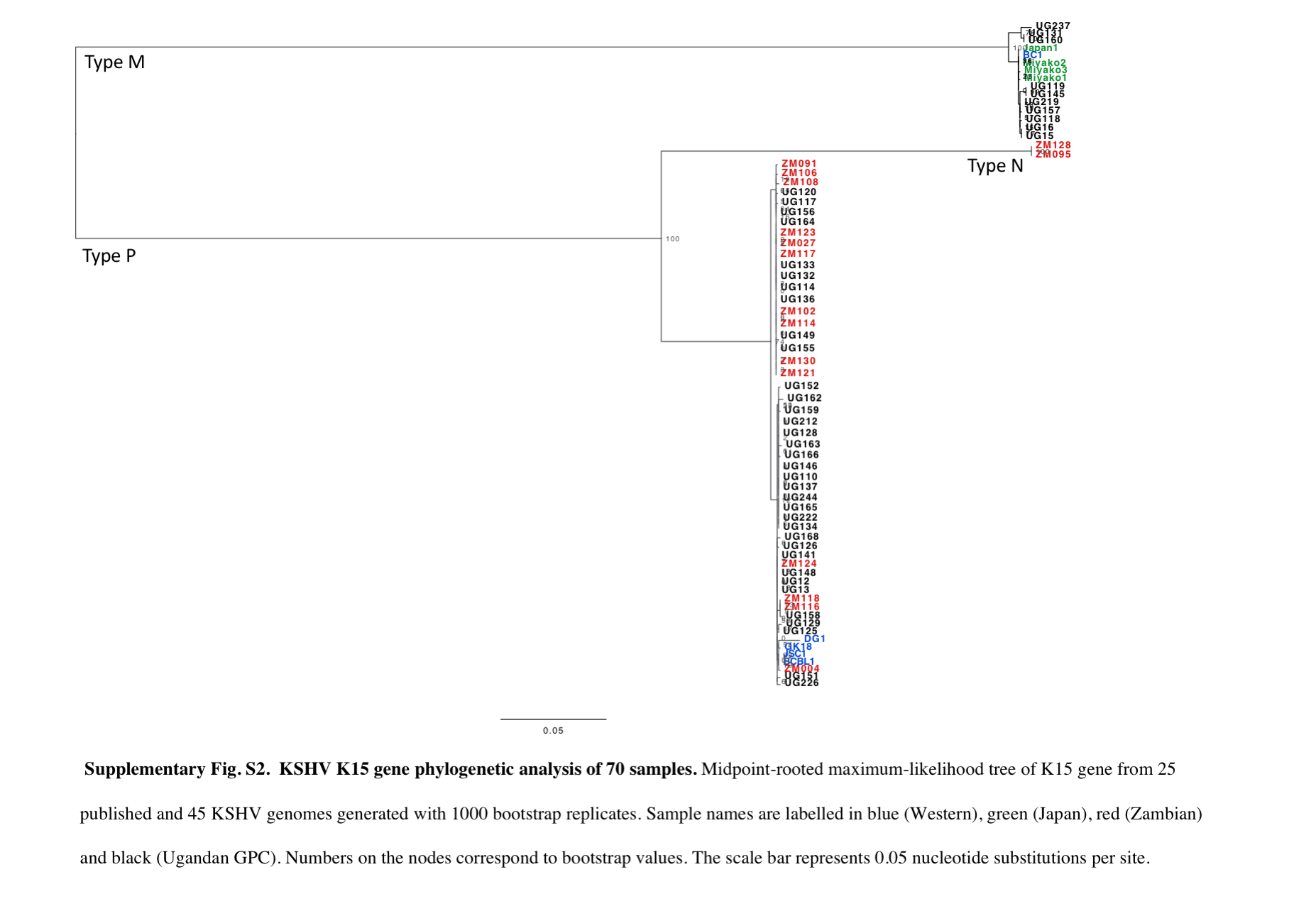

Supplement: Supplementary Figure 2 [file jiy427_suppl_supplementary_figure2.png]

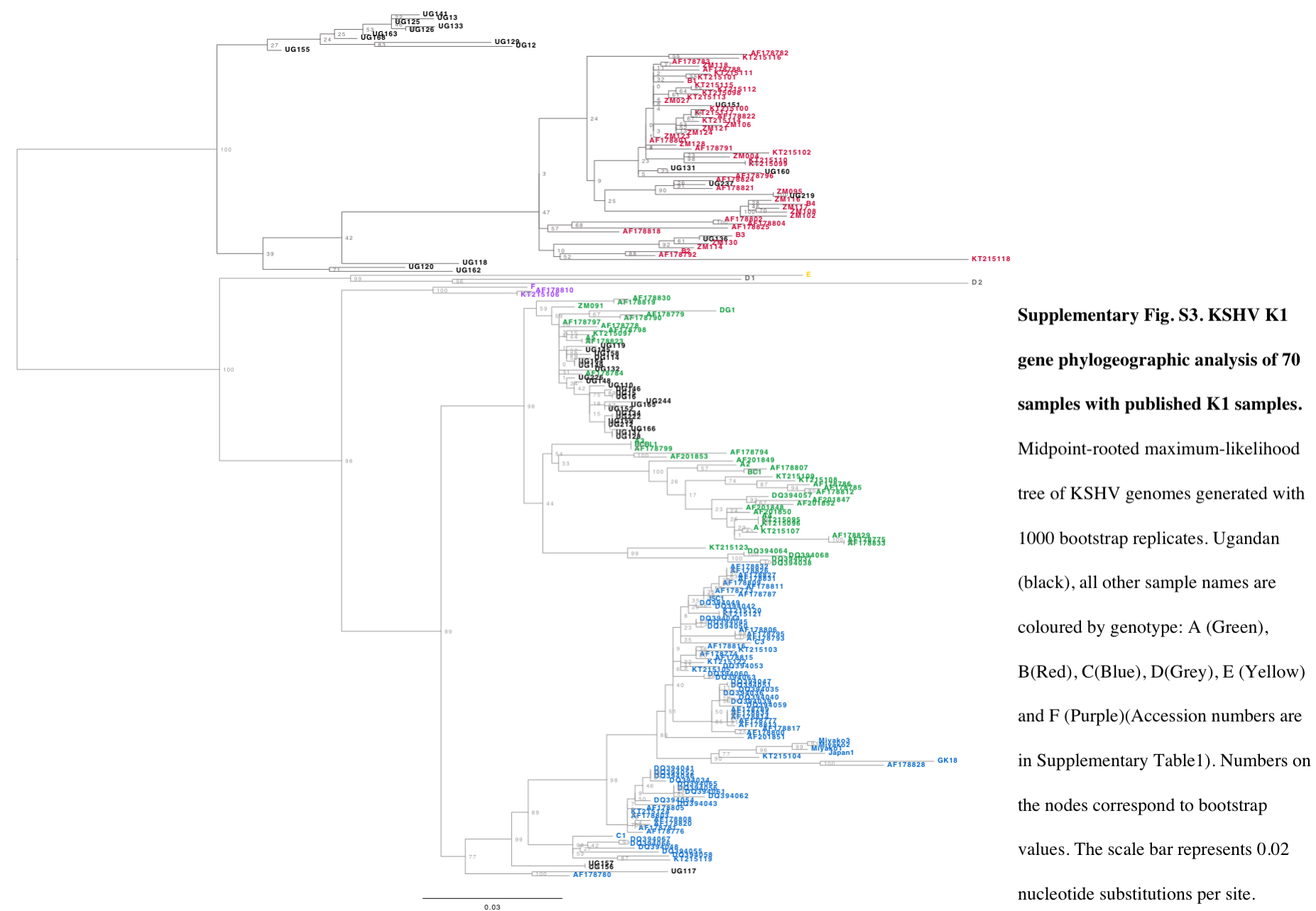

Supplement: Supplementary Figure 3 [file jiy427_suppl_supplementary_figure3.png]

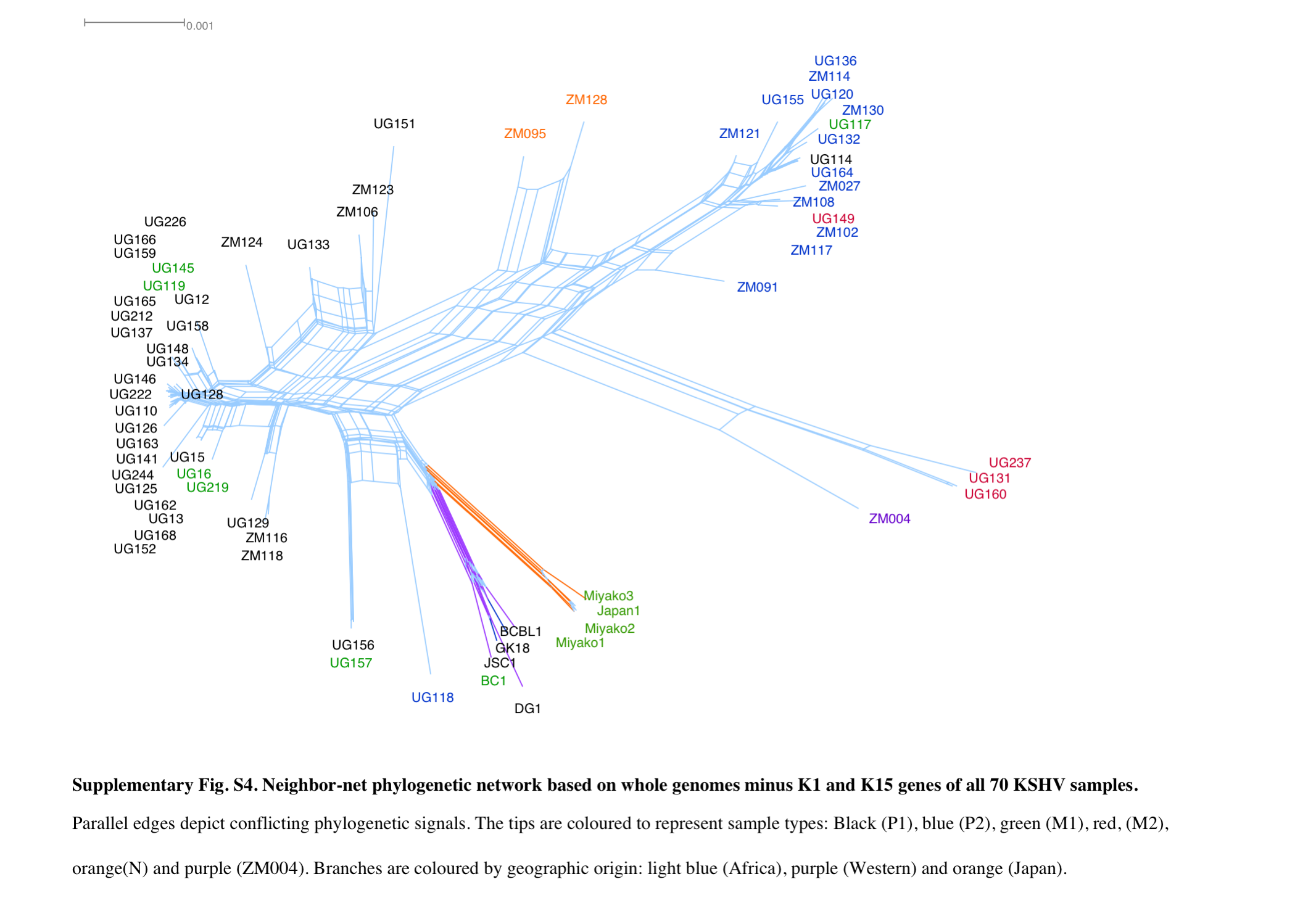

Supplement: Supplementary Figure 4 [file jiy427_suppl_supplementary_figure4.png]

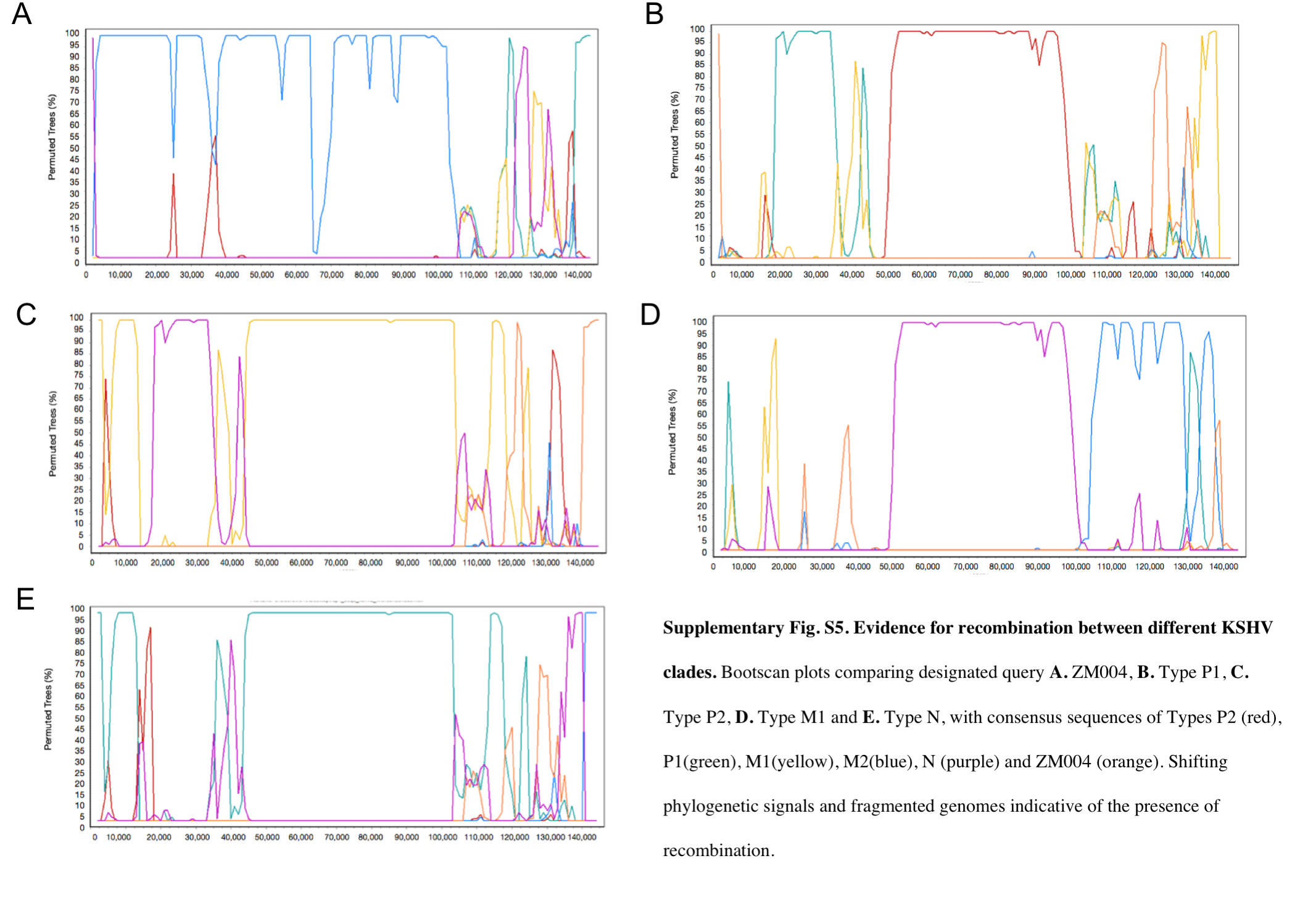

Supplement: Supplementary Figure5 [file jiy427_suppl_supplementary_figure5.png]
