# Supplementary material for: Genome-Wide Sequence Analysis of Kaposi Sarcoma-Associated Herpesvirus Shows Diversification Driven by Recombination
Source: J Infect Dis. 2018 Jul 14;218(11):1700–10. doi: 10.1093/infdis/jiy427 (PMC6195662; doi:10.1093/infdis/jiy427)
Supplement: Supplementary Table 1 [file jiy427_suppl_supplementary_table_1.docx]

**Supplementary Table 1. Published K1 genotypes used in this study**

| Accession Number | K1 Type | Ref |
| --- | --- | --- |
| AF133038 | A1 | [1] |
| AF130305 | A2 | [2] |
| U86667 | A3 | [3] |
| AF133039 | A4 | [1] |
| AF178823 | A5 | [4] |
| AF133040 | B1 | [1] |
| AY042947 | B2 | [5] |
| AY042941 | B3 | [5] |
| DQ309754 | B4 | [6] |
| AF133041 | C1 | [1] |
| AF133042 | C3 | [1] |
| AF133043 | D1 | [1] |
| AF133044 | D2 | [1] |
| AF220292 | E | [7] |
| FJ884616 | F | [8] |
| AF171056 | B2 | [1] |
| AF171057 | A5 | [1] |
| AF171058 | A5 | [1] |
| AF171059 | A5 | [1] |
| AF178773 | C3 | [1] |
| AF178774 | C3 | [1] |
| AF178775 | A1 | [1] |
| AF178776 | C7 | [1] |
| AF178777 | C3 | [1] |
| AF178778 | A5 | [1] |
| AF178779 | A5 | [1] |
| AF178780 | C | [1] |
| AF178781 | C7 | [1] |
| AF178782 | B1 | [1] |
| AF178783 | B1 | [1] |
| AF178784 | A5 | [1] |
| AF178785 | A | [1] |
| AF178786 | A2 | [1] |
| AF178787 | C | [1] |
| AF178788 | B1 | [1] |
| AF178789 | C | [1] |
| AF178790 | A5 | [1] |
| AF178791 | B1 | [1] |
| AF178792 | B2 | [1] |
| AF178793 | C3 | [1] |
| AF178794 | A | [1] |
| AF178795 | C3 | [1] |
| AF178796 | B1 | [1] |
| AF178797 | A5 | [1] |
| AF178798 | A5 | [1] |
| AF178799 | A3 | [1] |
| AF178800 | C3 | [1] |
| AF178801 | B1 | [1] |
| AF178803 | C7 | [1] |
| AF178804 | B2 | [1] |
| AF178805 | C7 | [1] |
| AF178806 | C3 | [1] |
| AF178807 | A2 | [1] |
| AF178808 | C7 | [1] |
| AF178810 | F | [1] |
| AF178811 | C3 | [1] |
| AF178812 | A | [1] |
| AF178818 | B2 | [1] |
| AF178819 | B3 | [1] |
| AF178820 | C | [1] |
| AF178821 | C | [1] |
| AF178822 | B1 | [1] |
| AF178823 | A5 | [1] |
| AF178824 | B1 | [1] |
| AF178825 | A5 | [1] |
| AF178828 | A | [1] |
| AF178829 | A | [1] |
| KT215124 | C7 | [9] |
| KT215123 | C5 | [9] |
| KT215122 | C3 | [9] |
| KT215121 | C3 | [9] |
| KT215120 | C3 | [9] |
| KT215119 | C2 | [9] |
| KT215118 | B2 | [9] |
| KT215117 | B1 | [9] |
| KT215116 | B1 | [9] |
| KT215115 | B1 | [9] |
| KT215114 | B1 | [9] |
| KT215113 | B1 | [9] |
| KT215112 | B1 | [9] |
| KT215111 | B1 | [9] |
| KT215110 | B1 | [9] |
| KT215109 | A2 | [9] |
| KT215108 | A2 | [9] |
| KT215107 | A1 | [9] |
| KT215106 | F | [9] |
| KT215105 | C3 | [9] |
| KT215104 | C3 | [9] |
| KT215103 | C3 | [9] |
| KT215102 | B1 | [9] |
| KT215101 | B1 | [9] |
| KT215100 | B1 | [9] |
| KT215099 | B1 | [9] |
| KT215098 | B1 | [9] |
| KT215097 | A5 | [9] |
| KT215096 | A4 | [9] |
| KT215095 | A4 | [9] |
| DQ394034 | C7 | [10] |
| DQ394035 | C3 | [10] |
| DQ394036 | C3 | [10] |
| DQ394037 | C5 | [10] |
| DQ394038 | C5 | [10] |
| DQ394039 | C3 | [10] |
| DQ394040 | C3 | [10] |
| DQ394041 | C7 | [10] |
| DQ394046 | C7 | [10] |
| DQ394042 | C3 | [10] |
| DQ394049 | C3 | [10] |
| DQ394050 | C3 | [10] |
| DQ394043 | C7 | [10] |
| DQ394044 | C3 | [10] |
| DQ394045 | C3 | [10] |
| DQ394047 | C3 | [10] |
| DQ394048 | C2 | [10] |
| DQ394051 | C3 | [10] |
| DQ394052 | C7 | [10] |
| DQ394053 | C3 | [10] |
| DQ394054 | C7 | [10] |
| DQ394055 | C1 | [10] |
| DQ394056 | C7 | [10] |
| DQ394057 | A | [10] |
| DQ394058 | C2 | [10] |
| DQ394059 | C3 | [10] |
| DQ394060 | C3 | [10] |
| DQ394061 | C7 | [10] |
| DQ394062 | C7 | [10] |
| DQ394063 | C3 | [10] |
| DQ394064 | C5 | [10] |
| DQ394065 | C7 | [10] |
| DQ394066 | C2 | [10] |
| DQ394067 | C2 | [10] |
| DQ394068 | C5 | [10] |

REFERENCES

1. Nicholas J, Zong JC, Alcendor DJ, et al. Novel organizational features, captured cellular genes, and strain variability within the genome of KSHV/HHV8. J Natl Cancer Inst Monogr **1998**:79-88.

2. Cook PM, Whitby D, Calabro ML, et al. Variability and evolution of Kaposi's sarcoma-associated herpesvirus in Europe and Africa. International Collaborative Group. AIDS **1999**; 13:1165-76.

3. Lagunoff M, Ganem D. The structure and coding organization of the genomic termini of Kaposi's sarcoma-associated herpesvirus. Virology **1997**; 236:147-54.

4. Lacoste V, Judde JG, Briere J, et al. Molecular epidemiology of human herpesvirus 8 in africa: both B and A5 K1 genotypes, as well as the M and P genotypes of K14.1/K15 loci, are frequent and widespread. Virology **2000**; 278:60-74.

5. Kakoola DN, Sheldon J, Byabazaire N, et al. Recombination in human herpesvirus-8 strains from Uganda and evolution of the K15 gene. J Gen Virol **2001**; 82:2393-404.

6. White T, Hagen M, Gudza I, et al. Genetic diversity of the Kaposi's sarcoma herpesvirus K1 protein in AIDS-KS in Zimbabwe. Journal of clinical virology : the official publication of the Pan American Society for Clinical Virology **2008**; 42:165-71.

7. Biggar RJ, Whitby D, Marshall V, Linhares AC, Black F. Human herpesvirus 8 in Brazilian Amerindians: a hyperendemic population with a new subtype. The Journal of infectious diseases **2000**; 181:1562-8.

8. Tornesello ML, Biryahwaho B, Downing R, et al. Human herpesvirus type 8 variants circulating in Europe, Africa and North America in classic, endemic and epidemic Kaposi's sarcoma lesions during pre-AIDS and AIDS era. Virology **2010**; 398:280-9.

9. Tozetto-Mendoza TR, Ibrahim KY, Tateno AF, et al. Genotypic distribution of HHV-8 in AIDS individuals without and with Kaposi sarcoma: Is genotype B associated with better prognosis of AIDS-KS? Medicine (Baltimore) **2016**; 95:e5291.

10. Duprez R, Hbid O, Afonso P, et al. Molecular epidemiology of the HHV-8 K1 gene from Moroccan patients with Kaposi's sarcoma. Virology **2006**; 353:121-32.
