# Supplementary material for: Genome-Wide Sequence Analysis of Kaposi Sarcoma-Associated Herpesvirus Shows Diversification Driven by Recombination
Source: J Infect Dis. 2018 Jul 14;218(11):1700–10. doi: 10.1093/infdis/jiy427 (PMC6195662; doi:10.1093/infdis/jiy427)
Supplement: Supplementary Table 2 [file jiy427_suppl_supplementary_table_2.docx]

**Supplementary Table 1 Summary of KSHV genome sequencing results**

| **Name** | **Village No.** | **Sex^a^** | **Age** | **HIV ^b^** | **Viral Load (Copies/ml)** | **Mean Depth** | **Mapped Reads (%)** |  |
| --- | --- | --- | --- | --- | --- | --- | --- | --- |
| UG110 | 12 | 1 | 40 | 0 | 2980 | 25X | 23.3 |  |
| UG114 | 14 | 1 | 18 | 0 | 45200 | 25X | 33.5 |  |
| UG117 | 14 | 2 | 43 | 0 | 24700 | 25X | 35.1 |  |
| UG118 | 14 | 1 | 21 | 0 | 6000 | 25X | 8.6 |  |
| UG119 | 14 | 2 | 36 | 0 | 11900 | 25X | 19.6 |  |
| UG12 | 13 | 2 | 53 | 1 | 33600 | 400X | 40.3 |  |
| UG120 | 14 | 1 | 77 | 0 | 15500 | 25X | 15.6 |  |
| UG125 | 14 | 2 | 20 | 0 | 15300 | 25X | 35.2 |  |
| UG126 | 14 | 1 | 18 | 0 | 535000 | 1000X | 70.8 |  |
| UG128 | 15 | 1 | 21 | 0 | 67700 | 500X | 65.2 |  |
| UG129* | 15 | 2 | 50 | 0 | 23400 | 25X | 42.6 |  |
| UG13 | 12 | 2 | 16 | 0 | 16000 | 200X | 29.7 |  |
| UG131* | 15 | 2 | 52 | 0 | 93700 | 500X | 62.2 |  |
| UG132 | 15 | 2 | 46 | 0 | 18600 | 25X | 11.7 |  |
| UG133 | 15 | 2 | 25 | 0 | 26600 | 25X | 35.7 |  |
| UG134 | 15 | 2 | 19 | 0 | 5730 | 25X | 12.5 |  |
| UG136 | 15 | 1 | 79 | 0 | 4730 | 25X | 15.6 |  |
| UG137 | 15 | 1 | 30 | 0 | 13000 | 25X | 22.5 |  |
| UG141 | 15 | 1 | 23 | 0 | 24200 | 25X | 34.4 |  |
| UG145 | 15 | 1 | 59 | 0 | 7260 | 25X | 21.8 |  |
| UG146 | 15 | 2 | 42 | 0 | 20200 | 25X | 26.1 |  |
| UG148 | 15 | 2 | 17 | 0 | 1560 | 25X | 10.7 |  |
| UG149 | 15 | 2 | 86 | 0 | 53500 | 25X | 40.6 |  |
| UG15 | 13 | 1 | 85 | 0 | 7040 | 25X | 21.4 |  |
| UG151 | 15 | 1 | 79 | 0 | 5630 | 25X | 11.4 |  |
| UG152 | 15 | 1 | 19 | 0 | 3720 | 25X | 1.7 |  |
| UG155 | 16 | 2 | 29 | 0 | 6410 | 25X | 30.3 |  |
| UG156 | 16 | 2 | 31 | 1 | 104000 | 1000X | 36.6 |  |
| UG157 | 16 | 2 | 30 | 1 | 37000 | 1000X | 34.9 |  |
| UG158 | 16 | 2 | 37 | 0 | 9140 | 20X | 1.9 |  |
| UG159 | 16 | 2 | 56 | 0 | 8960 | 25X | 15.6 |  |
| UG16 | 13 | 2 | 69 | 0 | 2150 | 25X | 13.9 |  |
| UG160 | 16 | 2 | 40 | 0 | 8670 | 25X | 19.8 |  |
| UG162 | 16 | 1 | 22 | 0 | 9590 | 25X | 30.2 |  |
| UG163 | 16 | 1 | 30 | 0 | 22000 | 25X | 23.9 |  |
| UG164 | 16 | 1 | 21 | 0 | 135000 | 750X | 76.8 |  |
| UG165 | 16 | 1 | 72 | 0 | 15200 | 25X | 15.2 |  |
| UG166 | 16 | 1 | 18 | 0 | 9140 | 25X | 24.9 |  |
| UG168 | 17 | 2 | 50 | 1 | 24900 | 25X | 26.5 |  |
| UG212 | 17 | 1 | 20 | 0 | 31900 | 25X | 16.9 |  |
| UG219 | 17 | 2 | 42 | 0 | 16800 | 25X | 11 |  |
| UG222 | 18 | 1 | 22 | 0 | 19900 | 25X | 10 |  |
| UG226 | 18 | 1 | 51 | 1 | 8220 | 20X | 1.5 |  |
| UG237 | 19 | 1 | 75 | 0 | 11700 | 25X | 18 |  |
| UG244 | 19 | 2 | 43 | 0 | 2910 | 20X | 5.3 |  |
| ^a^ Sex: 1=Male, 2=Female | | | | | | | | |
| ^b^ HIV Status: 0=Negative, 1=Positive | | | | | | | | |
| *Belong to the same household | | | | | | | | |
